# Quantitative parameter analysis of pretreatment dual-energy computed tomography in nasopharyngeal carcinoma cervical lymph node characteristics and prediction of radiotherapy sensitivity

**DOI:** 10.1186/s13014-024-02468-9

**Published:** 2024-06-26

**Authors:** Zhiru Li, Chao Li, Liyan Li, Dong Yang, Shuangyue Wang, Junmei Song, Muliang Jiang, Min Kang

**Affiliations:** 1 Department of Oncology, Sichuan Provincial People’s Hospital·Qionglai Medical Center Hospital, Chengdu, Sichuan People’s Republic of China; 2 Department of Radiation Oncology, The First Affiliated Hospital of Guangxi Medical University, No. 6, Shuangyong Road, Nanning, Guangxi, Guangxi 530021 People’s Republic of China; 3 Guangxi Tumor Radiation Therapy Clinical Medical Research Center, Nanning, Guangxi People’s Republic of China; 4 Department of Obstetrics and Gynecology, Sichuan Provincial People’s Hospital·Qionglai Medical Center Hospital, Chengdu, Sichuan People’s Republic of China; 5 Department of Radiology, The First Affiliated Hospital of Guangxi Medical University, Nanning, Guangxi People’s Republic of China

**Keywords:** Dual-energy CT, Nasopharyngeal carcinoma, Lymph nodes, Radiotherapy sensitivity

## Abstract

**Background:**

Treatment efficacy may differ among patients with nasopharyngeal carcinoma (NPC) at similar tumor–node–metastasis stages. Moreover, end-of-treatment tumor regression is a reliable indicator of treatment sensitivity. This study aimed to investigate whether quantitative dual-energy computed tomography (DECT) parameters could predict sensitivity to neck–lymph node radiotherapy in patients with NPC.

**Methods:**

Overall, 388 lymph nodes were collected from 98 patients with NPC who underwent pretreatment DECT. The patients were divided into complete response (CR) and partial response (PR) groups. Clinical characteristics and quantitative DECT parameters were compared between the groups, and the optimal predictive ability of each parameter was determined using receiver operating characteristic (ROC) analysis. A nomogram prediction model was constructed and validated using univariate and binary logistic regression.

**Results:**

DECT parameters were higher in the CR group than in the PR group. The iodine concentration (IC), normalized IC, Mix-0.6, spectral Hounsfield unit curve slope, effective atomic number, and virtual monoenergetic images were significantly different between the groups. The area under the ROC curve of the DECT parameters was 0.73–0.77. Based on the binary logistic regression, a column chart was constructed using 10 predictive factors, including age, sex, N stage, maximum lymph node diameter, arterial phase NIC, venous phase NIC, λHU and spectral Hounsfield units at 70 keV. The area under the ROC curve value of the constructed model was 0.813, with a sensitivity and specificity of 85.6% and 81.3%, respectively.

**Conclusion:**

Quantitative DECT parameters could effectively predict the sensitivity of NPC to radiotherapy. Therefore, DECT parameters and NPC clinical features can be combined to construct a nomogram with high predictive power and used as a clinical analytical tool.

**Supplementary Information:**

The online version contains supplementary material available at 10.1186/s13014-024-02468-9.

## Introduction

Nasopharyngeal carcinoma (NPC) is a common malignant tumor of the neck with a high incidence in Southeast Asia [1]. Early radiotherapy alone can successfully control tumors, whereas concurrent chemotherapy is recommended for locally advanced NPC. The 5-year overall survival (OS) rate is reportedly 85% [2–4]. Recurrence and/or metastasis are the main causes of treatment failure [5, 6]. The tumor–node–metastasis (TNM) staging system is a key factor determining the treatment regimen and prognosis of distant metastasis [7]. However, distant metastases differ significantly among patients with similar TNM stages [8], and N staging is not considered comprehensive or sufficiently accurate [9, 10].

As radiotherapy and chemotherapy are the main treatment for NPC rather than surgery, clinical examinations cannot be used to accurately evaluate pathological specimens of NPC lymph nodes. Magnetic resonance imaging (MRI) has been widely used to measure lymph node sizes because of its excellent ability to measure soft tissue components [11]. However, false-positive or -negative results often occur because large lymph nodes may be reactive, and small lymph nodes may also contain metastases [12–14]. Functional MRI techniques, such as diffusion-weighted imaging, have limited sensitivity (71.0–95.5%) and specificity (72.7–95.0%) in differentiating solid tumors [14, 15]. In addition to the MRI diagnostic criteria for retropharyngeal and cervical lymph node metastases in the international consensus guidelines [16], lymph nodes with high radiotherapy and chemotherapy sensitivity should be considered positive nodes. Understanding lymph node characteristics and improving the control of metastatic cervical lymph nodes are helpful for prolonging patient survival.

Dual-energy computed tomography (DECT) is an advanced CT scanning technology used to, in addition to the traditional single-energy CT scan, perform reconstruction and quantitative analysis, improve tumor visibility, delineate tumor boundaries, and determine critical structures in head and neck imaging [17]. Low-energy virtual monoenergetic images (VMIs) can be used to improve pathological lymph node visibility, similar to primary tumors [18]. Studies have reported different quantitative DECT parameters that can be used to describe lymph nodes, with significant differences observed in the quantitative parameters obtained from the spectral Hounsfield unit attenuation curve slope and iodine maps (iodine content) of lymph nodes [19]. These studies indicate that quantitative analysis helps identify lymph nodes with different pathological features.

Studies on the prediction of neck-lymph node radiotherapy sensitivity to NPC based on DECT quantitative parameters are lacking. Therefore, this study aimed to investigate whether quantitative DECT parameters can predict cervical-lymph node radiotherapy sensitivity to NPC, construct a nomogram by combining clinical and pathological factors with quantitative DECT parameters, evaluate the robustness of this new clinical predictive model, and provide new ideas for clinical diagnosis and treatment of NPC.

## Materials and methods

### Patient population

This study was performed in accordance with the tenets of the Declaration of Helsinki (revised in 2013) and was approved by the Medical Ethics Committee of the First Affiliated Hospital of Guangxi Medical University. All patients signed an informed consent form after receiving a detailed explanation of the research. Between September 2021 and December 2022, 98 patients newly diagnosed with nasopharyngeal carcinoma underwent pretreatment DECT. The study included patients (I) with pathologically confirmed NPC; (II) who had not received radiotherapy or chemotherapy before surgery; and (III) without a history of iodine allergy or hyperthyroidism symptoms. The exclusion criteria were as follows: (1) incomplete clinical data; (2) poor image quality that could not be qualitatively or quantitatively analyzed; and (3) a history of tuberculosis, other head and neck malignancies, or lymphoma.

All patients underwent pretreatment dual-energy DECT and MRI scans 1–3 days before treatment with radical intensity-modulated radiation therapy and concurrent ± induction chemotherapy. Radiotherapy was administered according to Reports 83 of the International Commission on Radiation Units and Measurements (ICRU) and the expert consensus of the Radiation Treatment Oncology Organization Group (RTOG) 0225. GTVnx includes primary NPC foci and enlarged retropharyngeal lymph nodes, whereas GTVnd includes imaging and palpation findings of enlarged cervical lymph nodes. The high-risk clinical target volume (CTV1) was a 5–10-mm outward expansion of the GTVnx (or 2–3 mm if close to the brainstem or spinal cord) to cover the submicroscopic increase in the high-risk site and entire nasopharynx. The low-risk clinical target volume (CTV2) was a 5–10-mm outward expansion of CTV1 to include the foramen lacerum, sphenoid sinus, clivus, oval foramen, parapharyngeal space, pterygoid fossae, posterior parts of the nasal cavity, pterygopalatine fossae, and the lymph node drainage area in the neck. Treatment doses included PTVnx and PTVRPN (68–74 Gy), PTVND (66–70 Gy), PTV1 (60–66 Gy), and PTV2 (50–56 Gy). Five fractions/week and a total of 30–33 fractions were administered. All chemotherapy regimens were platinum-based (80–100 mg/m^2^) and administered once every 3 weeks, including radiotherapy alone, concurrent chemoradiotherapy (CCRT), and CCRT after induction chemotherapy. Clinical and pathological data, including age, sex, body mass index, TNM staging, comorbidities, Epstein–Barr virus (EBV) DNA levels, radiation and chemotherapy status, and lymph node characteristics, were collected.

### Data acquisition and image reconstruction

All patients were scanned using a DECT scanner (SOMATOM Definition Flash CT; Siemens, Erlangen, Germany). Patients were placed on a scanning bed and instructed to avoid swallowing, and scanning was performed from the base of the skull to the sternoclavicular joint. Scanning parameters were: tube voltages, 80 kV and 140 kV (with simultaneous application of CARE Dose 4D); reference tube current, 320 mA; collimation, 128 × 0.6 mm; rotation speed, 0.5 s/r; and pitch, 0.6. A sinogram affirmative reconstruction (SAFIRE) technique was used for the reconstruction. The reconstruction layer thickness was 1.5 mm, and the space between layers was 1 mm. A high-pressure syringe (85 mL; Nemoto Kyorindo Co. Ltd., Tokyo, Japan) was used to inject the contrast agent iopamidol (iopamidol 300; Bracco, Milan, Italy) intravenously through the median elbow at 1–1.5 mL/kg body weight and 3 mL/s. The common carotid artery was selected as the detection point, and the threshold was set at 100 HU. The scan delay period for the arterial and venous phases was 25 s and 50 s, respectively.

### DECT image analysis

The reconstructed DECT image data were postprocessed using a workstation (VB20A; Siemens), and energy spectrum curve characteristics were analyzed using the “Liver VNC,” “Single Energy+,” and “Rho/Z” functions. Under different single-energy conditions (40, 50, 60, 70, …, 120 keV) and the corresponding single-energy conditions, when the image contrast-to-noise ratio value was the highest, the region of interest (ROI) was selected from the largest lymph node of each patient, and the CT value was calculated. The ROI included as many solid parts as possible, and each lesion was measured thrice in a blinded manner by three deputy chief physicians with over 10 years of experience in CT diagnosis. The iodine concentration (IC), effective atomic number(Z_eff_), spectral Hounsfield units at 40–100 keV (10-keV intervals), linear blending images with a blending ratio of 0.6 (Mix-0.6), and electron cloud density (Rho) of each lesion were recorded. The normalized IC (NIC) was calculated as IC(lesion)/IC(Common Carotid artery), and the spectral Hounsfield unit curve slope (λhu) was calculated as λhu = (HU40 keV-HU70 keV)/30 keV.

### Clinical evaluation

According to the Response Evaluation Criteria in Solid Tumors version 1.1 [20], responses were divided into complete response (CR; lesion disappearance), partial response (PR; at least 30% reduction in nodal diameter based on baseline diameter), stable response (< 30% reduction in nodal diameter), and progression (> 20% increase in nodal diameter). Lesion changes in completely and partially responsive patients were repeatedly confirmed. The patients were divided into CR and PR groups (partial response + stable response) according to therapeutic effects.

The TNM staging criteria for tumors were adopted in the 2017 International Alliance Against Cancer/American Joint Committee on Cancer (AJCC) 8th edition TNM staging standards [21]. The nodal division was determined according to the 2013 international consensus guidelines [16, 22]. Diagnostic MRI criteria for metastatic lymphadenopathy included [23] (1) the smallest lymph node diameter in the largest cross-sectional image is ≥ 10 mm; (2) lymph nodes of any size with central necrosis or a contrast-enhancing rimor exocapsular invasion; (3) lymph node grouping (presence of ≥ 3 contiguous and confluent lymph nodes, each with MID 8–10 mm); (4) the maximum transverse diameter of retropharyngeal lymph node is ≥ 5 mm. All included lymph nodes were positive according to MRI diagnostic criteria.

According to the lymph node location, patients were further divided into groups based on regions bounded by the hyoid body and the lower margin of cricoid cartilage: the upper cervical lymph node group (UNP), in which the lymph nodes were located in the retropharyngeal, I, and II regions; the middle cervical lymph node group (MNP), in which the lymph nodes were located in the upper part of the III, VA, and above VI regions; and the lower cervical lymph node group (LNP), where positive lymph nodes were located in the IV, superior clavicular fossa, and inferior portions of the VB and subarea VI regions.

### Statistical analysis

SPSS 26.0 (IBM Corp.) software was used for statistical analysis. Count data are expressed as percentages (%), and groups were compared using the χ^2^ test. Measurement data are expressed as mean ± standard deviation (x ± s), and the groups were compared using an independent sample t-test/Mann‒Whitney U-test (depending on the normality of the data distribution). Intra- and interobserver agreements were assessed using intragroup correlation coefficients in relation to quantitative parameters. Logistic regression analysis was used to fit the significant parameters. Receiver operating characteristic (ROC) analysis was used to calculate the area under the ROC curve (AUC) to evaluate the diagnostic value of the quantitative DECT parameters for radiotherapy sensitivity. The cutoff value was determined using the maximum Youden index, and the sensitivity, specificity, and AUC were calculated according to the optimal cutoff value. Logistic regression analysis was used to fit the significant parameters of single factors, and independent prognostic factors were determined to construct a nomogram, decision curve, and calibration plot for model evaluation. The open-source statistical environments R (version 4.3.0, available at www.r-project.org) and the “rms”, “foreign”, “rio” and “roc” packages were used for statistical analysis. The threshold for statistical significance was set at *p* < 0.05.

## Results

### Participants and lymph node characteristics

Overall, 98 patients, comprising 73 men and 25 women aged 30–70 years (mean age: 46.9 ± 10.9 years) with 388 lymph nodes, were included (Table 1). After radiotherapy, 285 and 103 lymph nodes were assigned to the CR and PR groups, respectively. The two groups differed significantly with respect to age, N stage, EBV DNA level, lymph node location, longest dimension (LD) and shortest dimension (SD) of lymph nodes, and MRI classification. However, no significant differences were observed with respect to sex; body mass index; the T, M or AJCC stage; or induction chemotherapy (Tables 2 and 3).

**Table 1:** Baseline characteristics of patients with nasopharyngeal carcinoma

Characteristics	Number	%
Total	98	100
Age (years)		
≤ 45	43	43.9
>45	56	57.1
Sex		
Male	73	74.5
Female	25	25.5
BMI(kg/m^2^)		
≤ 22.5	49	50
>22.5	49	50
AJCC stage		
II	2	2
III	23	23.5
IV	73	74.5
T stage		
T2	14	14.2
T3	37	37.8
T4	48	49
N stage		
N1	30	30.7
N2	22	22.4
N3	46	46.9
M stage		
M0	87	88.8
M1	11	11.2
Induction chemotherapy		
No	16	16.3
TP	43	43.9
GP	38	38.8
EBV DNA(copy/ml)		
≤ 2000	65	66.3
>2000	33	33.7

**Table 2:** Differences in lymph node response characteristics after radiotherapy

Characteristics	Total	CR group	PR group	*P* value
	[*n*]	[*n*]	[*n*]	
Total	388	285	103	
Age (years)				
≤ 45	162	104	58	0.002
>45	226	181	45	
Sex				
Male	319	248	71	<0.001
Female	69	37	32	
BMI(kg/m2)				
≤ 22.5	183	127	56	0.232
>22.5	205	158	47	
AJCC stage				
II				
III	60	38	22	0.155
IV	328	247	81	
T stage				
T2	47	36	11	0.663
T3	167	116	51	
T4	174	133	41	
N stage				
N1	53	35	18	0.047
N2	49	33	16	
N3	286	217	69	
M stage				
M0	342	250	92	0.911
M1	46	35	11	
Induction chemotherapy				
No	34	19	15	0.239
TP	164	116	48	
GP	190	150	40	
EBV(copy/ml)				
≤ 2000	203	139	64	0.047
>2000	185	146	39	

**Table 3:** Morphological characteristics of the nasopharyngeal carcinoma lymph nodes

Characteristics	Total	CR group	PR group	*P* value
	[*n*(%)]	[*n*(%)]	[*n*(%)]	
Total	388	285	103	
Lymph node location				
UNP	300	209	91	0.037
MNP	56	47	9	
LNP	32	29	3	
LD(cm)				
>1.5	143	92	51	0.023
1 ≥ D ≤ 1.5	164	125	39	
<1	81	68	13	
SD(cm)				
>1.5	128	83	45	0.043
1 ≥ D ≤ 1.5	179	134	45	
<1	81	68	13	

### Comparison of quantitative DECT parameters

The quantitative DECT parameters in the CR group were higher than those in the PR group. The two groups differed significantly with respect to the arterial and venous phase IC, NIC, Mix-0.6, λHU, Z_eff_, spectral Hounsfield units at 40–100 keV. Rho significantly differed between the arterial and venous phases (Table 4).

**Table 4:** Comparison of lymph node dual-energy computed tomography-derived quantitative parameters between the complete and partial response groups

DECT parameters	CR group	PR group	*P* value
Arterial phase			
IC(mg/mL)	1.82 ± 0.71	1.98 ± 0.61	0.035
NIC	0.15 ± 0.06	0.17 ± 0.05	0.039
Mix-0.6	76.94 ± 17.65	81.67 ± 14.28	0.007
λHU(HU/keV)	2.15 ± 0.79	2.36 ± 0.73	0.012
Z_eff_	8.48 ± 0.34	8.58 ± 0.29	0.008
Rho	37.51 ± 6.65	38.54 ± 5.03	0.102
40 keV(HU)	183.78 ± 45.72	199.94 ± 40.23	0.007
50 keV (HU)	131.96 ± 32.02	142.67 ± 32.47	0.006
60 keV(HU)	100.55 ± 25.72	108.03 ± 22.23	0.006
70 keV(HU)	81.22 ± 18.94	86.81 ± 16.06	0.004
80 keV(HU)	68.51 ± 14.77	73.13 ± 12.21	0.002
90 keV(HU)	60.37 ± 11.91	64.01 ± 9.76	0.003
100 keV(HU)	54.74 ± 10.11	57.79 ± 8.19	0.003
Venous phase			
IC(mg/mL)	2.29 ± 0.56	2.45 ± 0.46	0.005
NIC	0.42 ± 0.09	0.45 ± 0.08	0.001
Mix-0.6	86.92 ± 16.08	92.11 ± 11.05	0.003
λHU(HU/keV)	2.72 ± 0.66	2.91 ± 0.51	0.004
Z_eff_	8.72 ± 0.27	8.8 ± 0.21	0.003
Rho	38.63 ± 6.53	39.76 ± 5.17	0.077
40 keV(HU)	223.4 ± 46.07	237.31 ± 35.85	0.002
50 keV (HU)	157.61 ± 30.41	167.21 ± 23.73	0.001
60 keV(HU)	117.96 ± 20.95	124.76 ± 16.18	0.001
70 keV(HU)	93.85 ± 15.64	98.89 ± 12.06	0.001
80 keV(HU)	78.09 ± 12.22	81.95 ± 9.42	0.001
90 keV(HU)	67.55 ± 10.08	70.72 ± 7.68	0.001
100 keV(HU)	60.38 ± 8.75	63.01 ± 6.72	0.002

### Relationship between lymph node characteristics and quantitative DECT parameters

**Fig. 1:**
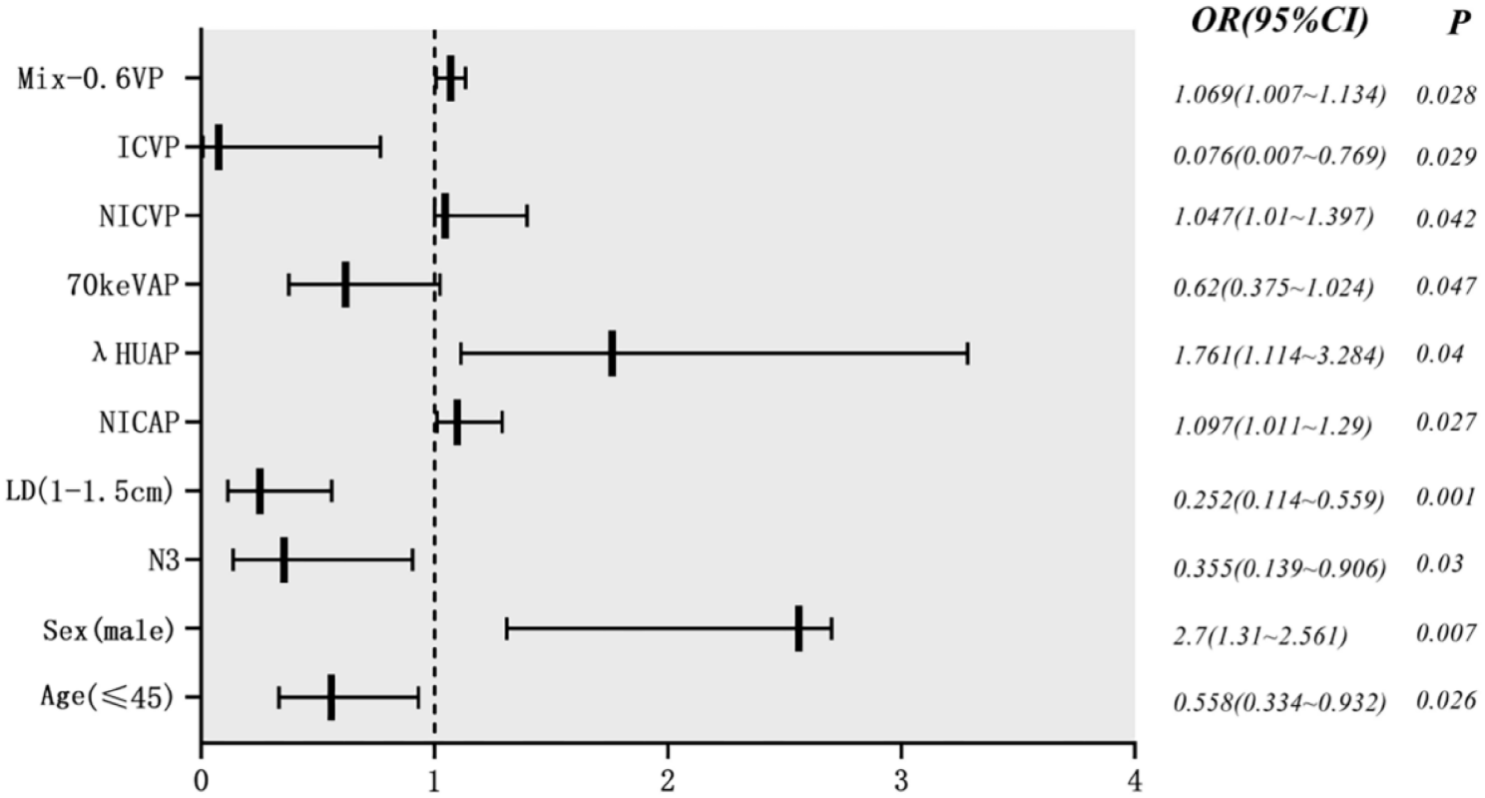
Multivariate analysis of clinical, pathological, morphological features and dual-energy parameters

**Fig. 2:**
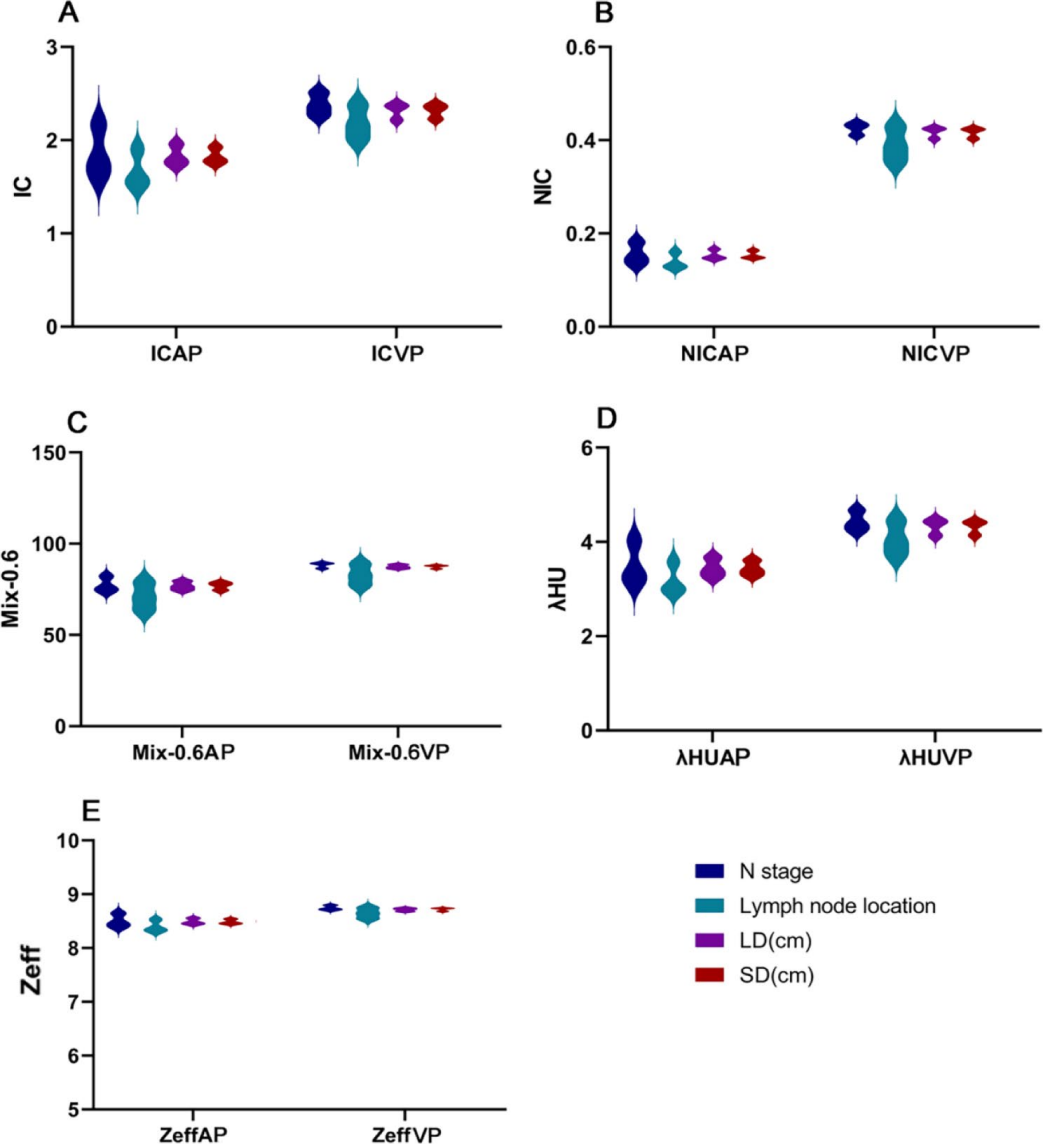
Relationship between lymph node characteristics and dual-energy parameters. **A** Comparison of IC in arterial phase and venous phase of lymph node characteristics; **B** Comparison of NIC in arterial phase and venous phase of lymph node characteristics; **C** Comparison of Mix-0.6 in arterial phase and venous phase of lymph node characteristics; **D** Comparison of λhu in arterial phase and venous phase of lymph node characteristics; **E** Comparison of Zeff in arterial phase and venous phase of lymph node characteristics

Binary logistic regression analyses showed that age, sex, N stage, LD, arterial phase NIC, arterial phase λHU, arterial phase spectral Hounsfield units at 70 keV, venous phase NIC, venous phase IC, and venous phase Mix-0.6 were significantly associated with radiotherapy sensitivity (Fig. 1). Further analysis showed that all DECT parameters in the arterial phase, including IC, NIC, Mix-0.6, λHU, and Z_eff_, were lower than those in the venous phase (Fig. 2). Subgroup analysis showed that there were significant differences in N stage, lymph node location, and LD among the parameter arterial phase IC, NIC, Mix-0.6, λHU, and Z_eff_. N stage, LD, and SD showed significant differences in venous phase IC and λHU; lymph node location showed significant differences in venous phase IC, NIC, Mix-0.6, and λHU showed significant differences in venous phase IC, NIC, Mix-0.6, and λHU (Supplementary Fig. 1).

### DECT parameters as predictors of therapeutic response

The cutoff values, AUC, accuracy, sensitivity, and specificity of the NIC, IC, Mix-0.6, λHU, Z_eff_, and spectral Hounsfield units at 70 keV during the arterial and venous phases are presented in Table 5. The AUC of all DECT parameters ranged from 0.73 to 0.77 (*P* < 0.001). The best cutoff values for the arterial phase were 0.16, 2.05, 87.35, 2.36, 8.65, and 86.70, whereas those for the venous phase were 0.41, 2.25, 88.65, 2.92, 8.73, and 92.70.

**Table 5:** The value of arterial and venous dual-energy parameters (IC, NIC, Mix-0.6, λHU, Z_eff_, and 70 keV) for radiotherapy sensitivity

	AUC	95%CI	Youden	Cutoff	Sensitivity	Specificity
Arterial phase						
NIC	0.73	(0.67–0.78)	0.34	0.16	0.64	0.70
IC	0.74	(0.68–0.79)	0.38	2.05	0.65	0.73
Mix-0.6	0.75	(0.69–0.79)	0.37	87.35	0.55	0.72
λHU	0.74	(0.68–0.79)	0.41	2.36	0.69	0.82
Zeff	0.74	(0.68–0.79)	0.37	8.65	0.60	0.77
70 keV	0.74	(0.69–0.79)	0.37	86.70	0.67	0.70
Venous phase						
NIC	0.73	(0.67–0.78)	0.40	0.41	0.86	0.64
IC	0.74	(0.69–0.79)	0.36	2.25	0.84	0.63
Mix-0.6	0.77	(0.72–0.82)	0.47	88.65	0.88	0.68
λHU	0.76	(0.71–0.81)	0.39	2.92	0.67	0.72
Zeff	0.75	(0.69–0.8)	0.37	8.73	0.83	0.65
70 keV	0.77	(0.72–0.81)	0.42	92.70	0.90	0.62

**Fig. 3:**
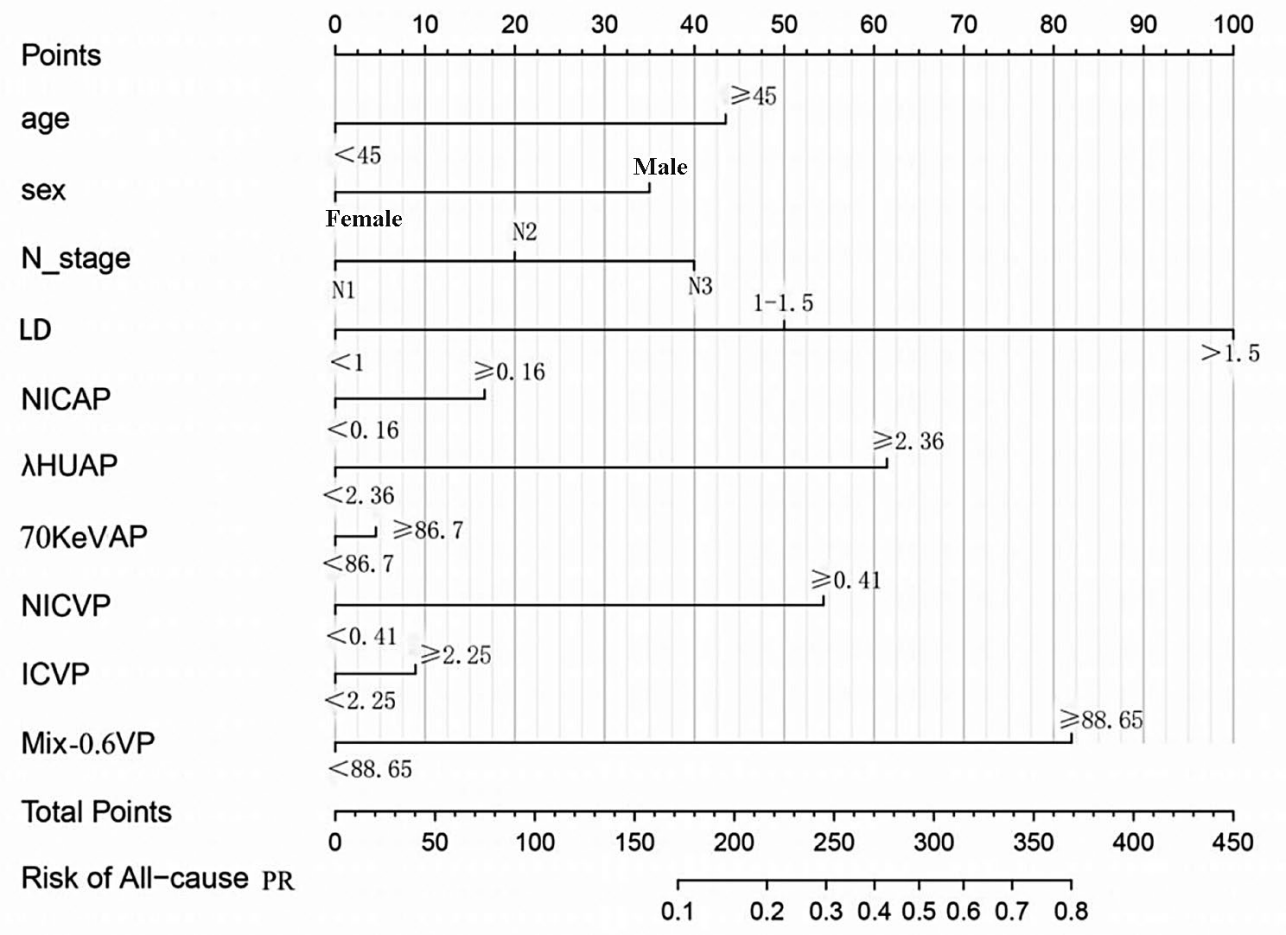
Nomogram to predict radiotherapy sensitivity of nasopharyngeal carcinoma

**Fig. 4:**
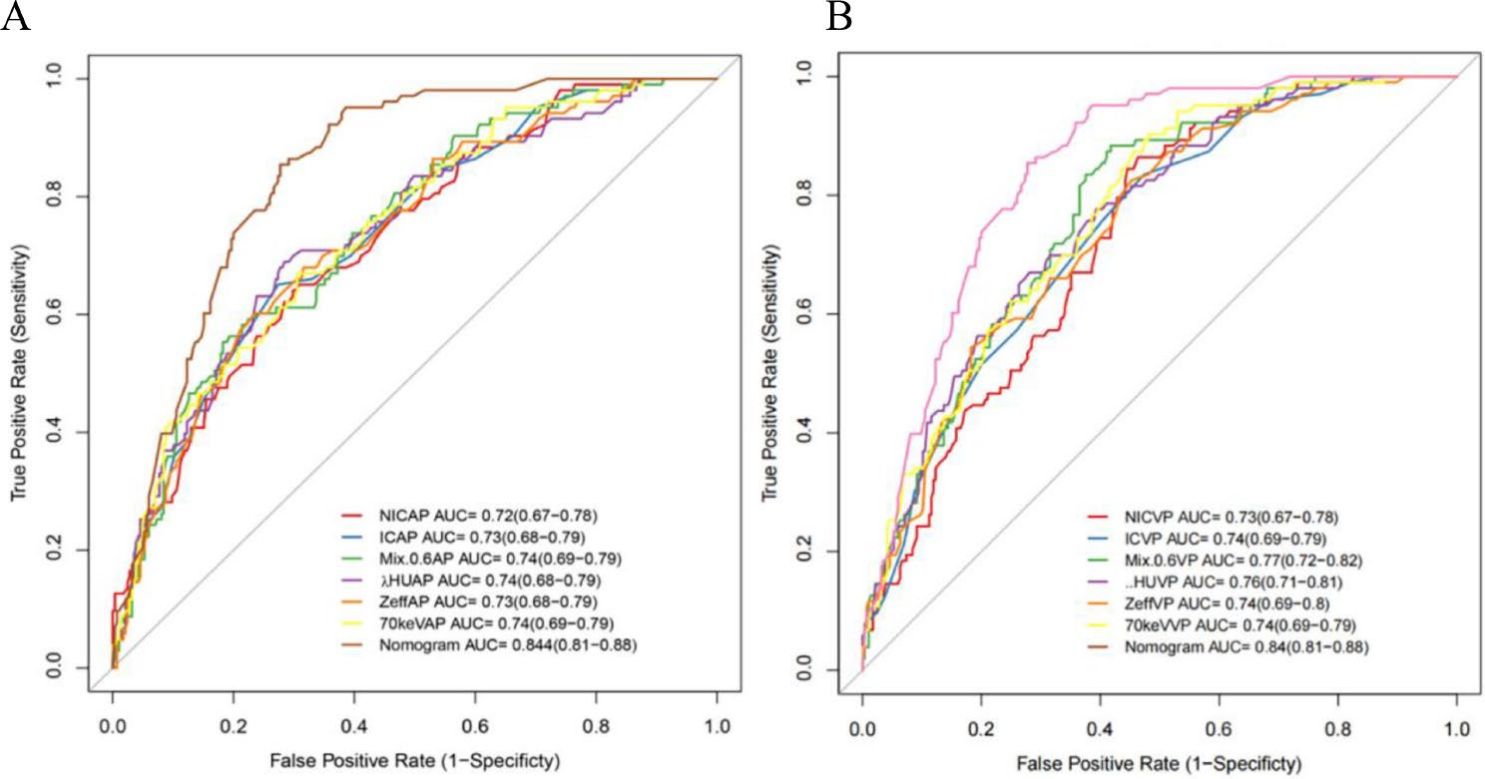
Receiver operating characteristic curves of the nomogram and dual-energy parameters.(**A**) ROC curves of the nomogram and arterial phase dual-energy parameters; (**B**) ROC curves of the nomogram and venous phase dual-energy parameters

**Fig. 5:**
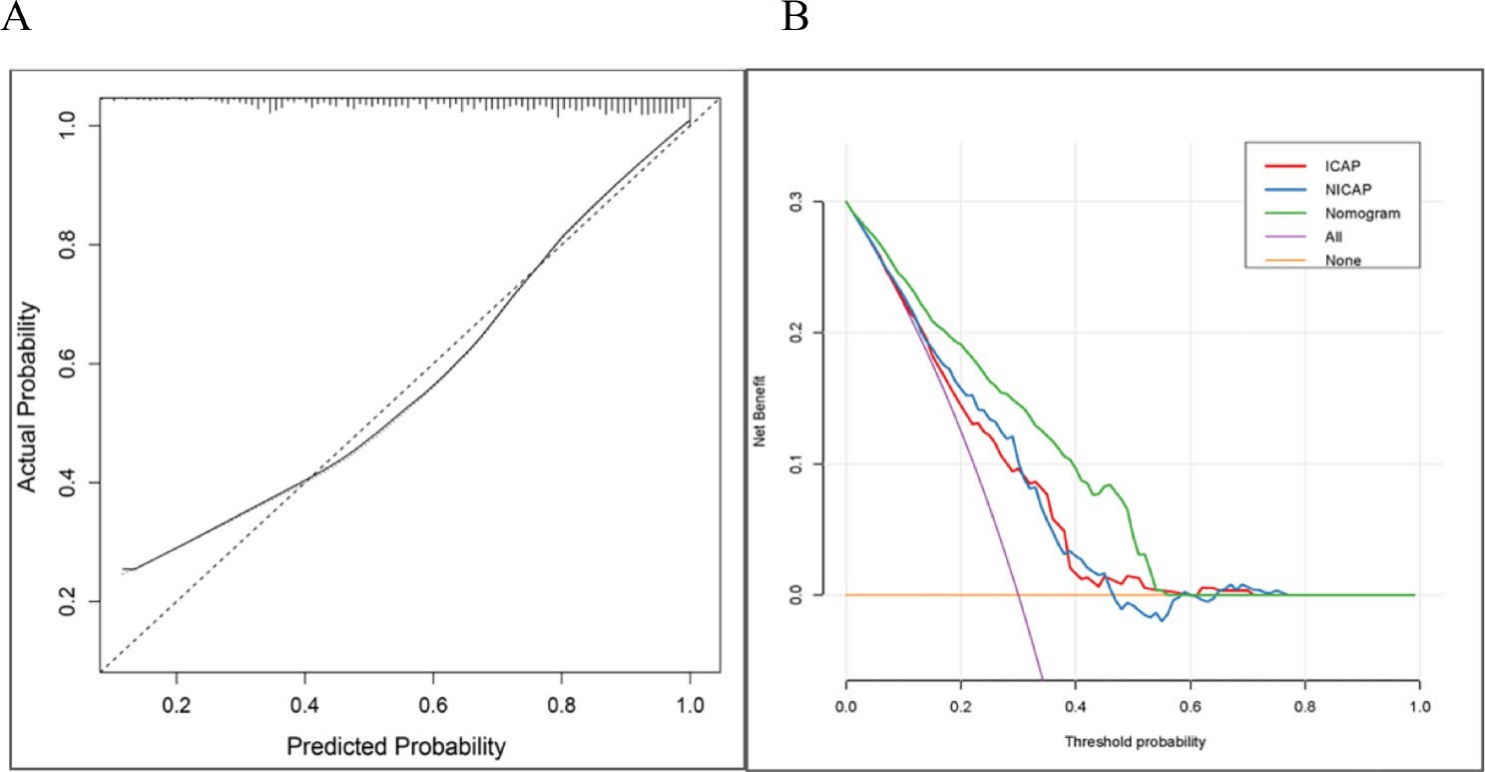
Calibration curves and decision curves for the nomogram. (**A**) Calibration curves of the nomogram to predict radiotherapy sensitivity; (**B**) Decision curves for the nomogram and dual-energy parameters

**Fig. 6:**
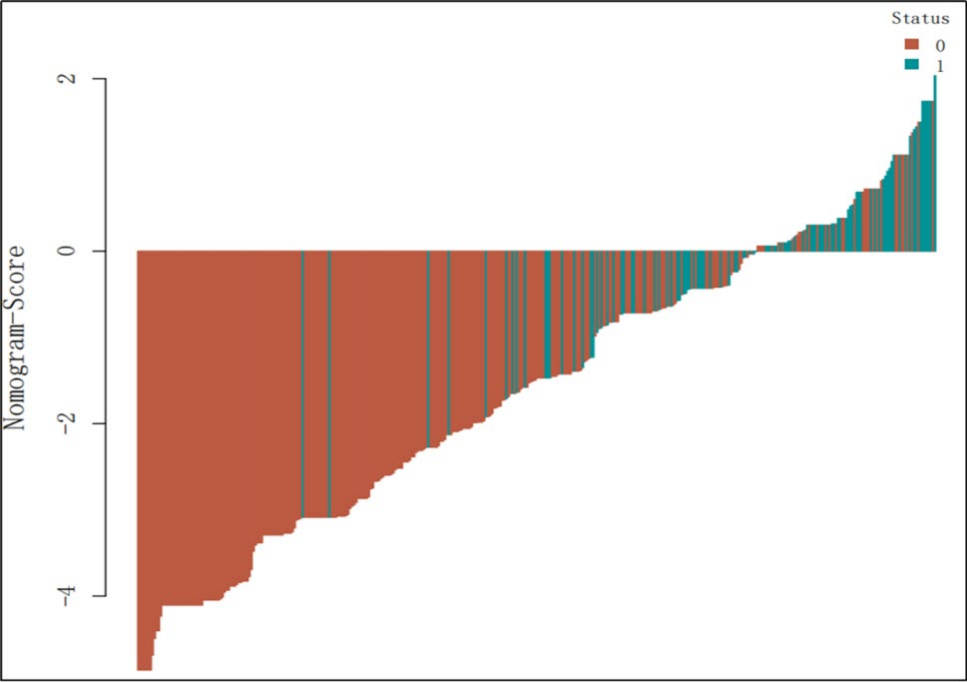
Waterfall plot for distribution of Nomo-score in LNM prediction

Clinicopathological factors and DECT parameters were included in the univariate and multivariate logistic regression analyses, and DECT parameters with significant differences were classified as categorical variables based on the cutoff values. Finally, a nomogram based on 10 predictors, including age, sex, N stage, arterial phase NIC, arterial phase λHU, arterial phase spectral Hounsfield units at 70 keV, venous phase NIC, venous phase IC, and venous phase Mix-0.6 was constructed (Fig. 3). The AUC value of the nomogram was 0.84 (95% confidence interval: 0.81–0.88), with sensitivity and specificity of 85.66% and 81.3%, respectively, indicating that the AUC of the nomogram was superior to that of a single DECT parameter and that the model had good predictive ability (Fig. 4). Additionally, the calibration curve showed that the predictive ability of the nomogram model was highly consistent with actual radiotherapy sensitivity (Fig. 5). Decision curves of IC, NIC, and the nomogram revealed that the net benefits of the nomogram were higher than those of the DECT parameters (Fig. 5). All patients were divided into high- and low-risk group of LNM according to the optimal nomo-score cutoff value of -1.238 (corresponding to a total of 234 points in nomogram). The waterfall diagram displayed the distribution of nomo-scores and the status of lymph node sensitivity to radiotherapy (Fig. 6).

## Discussion

Radiosensitivity is an important factor influencing the curative effects of NPC treatment. Gross tumor regression of primary tumors and/or metastatic lymph nodes at the end of intensity-modulated radiation therapy can be used to predict poor prognosis in patients with NPC [24, 25]. The difference in tissue radiosensitivity is the focus of research because of the significant differences in the clinical curative effect among patients receiving the same therapy and with similar EBV DNA levels, TNM stage, and pathological characteristics [26, 27].

Residual tumors appear after NPC radiotherapy, and tissue radiotherapy tolerance is related to several biological changes in the tumor and its microenvironment. The major factors are the degree of tissue hypoxia and the tumor molecular phenotype [26, 28, 29]. A close relationship exists between the blood supply and oxygen content of the tumor. The higher the blood supply and oxygen content, the higher the sensitivity to radiotherapy. The early prediction of radiosensitivity can lead to optimized treatment regimens and reduced medical costs. Conventional MRI often provides insufficient diagnostic evidence for the early prediction of the therapeutic efficacy of NPC treatment [30]. Recently, advanced MRI methods, including intravoxel incoherent motion diffusion-weighted, dynamic contrast-enhanced, and three-dimensional pseudo-continuous arterial spin labeling perfusion imaging, have been used to predict the therapeutic efficacy of NPC treatment [31, 32].

DECT can produce images of various substances (including mainly water, iodine, and calcium) separated into different material images, which can be quantitatively analyzed on different substrate images to obtain a tissue characteristic map that reflects the chemical composition of the tissue and quantifies the concentration of the component [33]. The distribution of IC in different tissues is reflected in the iodine characteristics of tumor angiogenesis [34–36]. Z_eff_ indicates the average atomic number of a mixture of composite substances in the tissue, which is related to the density of tumor cell components and tissue iodine content [37, 38]. Lower keV values can increase tumor visibility, whereas higher keV values can reduce beam hardening artifacts [39, 40]. λHU reflects the attenuation characteristics of the lesions under different energy conditions [41]. These DECT quantitative parameters can characterize tumor blood supply and reflect lymph node characteristics [36, 41–44].

To the best of our knowledge, this is the first study to use multiple quantitative DECT parameters to predict lymph node sensitivity to radiotherapy in patients with NPC. Our study showed that after radiotherapy, the DECT parameters of lymph node PR group were higher than those of CR group, and there were significant differences in IC, NIC, Mix-0.6,λHU, Zeff and spectral Hounsfield units at 40–100 keV in arterial and venous phases. It was suggested that lymph nodes in the PR group may have had higher blood vessel density. Multiple factors showed that arterial phase NIC, λHU, spectral Hounsfield units at 70 keV, venous phase NIC, IC, and Mix-0.6 were independent predictors.Some of the high-energy VMIs and Rho were not significantly different, consistent with the findings of Zhao et al. [41, 45, 46] but not with those of Liu et al. [47]. This may be due to differences in the characteristics of the primary tumor and lymph nodes and the increased difference in iodine deficiency intensity of the high-energy VMIs. The heterogeneity of lymph nodes may be better reflected in low-energy VMIs and iodine maps.

Furthermore, we found that quantitative DECT parameters, such as IC, NIC, Mix-0.6, λHU, and Z_eff_, were lower in the arterial phase than in the venous phase. The difference in iodine content parameters between the arterial and venous phases was greater, indicating a higher prediction, consistent with Qiu et al.’s study [46] on rectal cancer lymph nodes. There were also differences in the arterial and venous phase parameters with different N stages, and lymph node locations and lengths, which we considered to be correlated with the degree of tumor invasiveness in patients with different clinical characteristics. For example, patients with high clinical stage tended to have large tumors with internal necrosis and loss of blood supply to the tumor stroma.

Using DECT to predict radiotherapy sensitivity in NPC, we found that the AUCs of IC, NIC, Mix-0.6, λHU, Z_eff_ and spectral Hounsfield units at 70 keV were equivalent (0.73–0.77) and slightly higher in the arterial phase than in the venous phase. Wang et al. [36] reported that DECT could simultaneously provide multiple parameters reflecting tumor parenchyma and vasculature information, which can minimize the overlap of DECT-derived single parameters and improve the overall performance of solid tumors in the differential diagnosis. In this study, univariate and multivariate regression analyses were performed to estimate clinicopathological variables and DECT parameters, and a nomogram containing 10 independent factors was established. The nomogram showed that LD, arterial λHU, venous NIC, and Mix-0.6 were more effective in establishing a predictive model with a higher AUC value (0.84), sensitivity and specificity were 85.6% and 81.3%, respectively, can be used to develop individualized treatment plans for patients with NPC, and is useful for the early identification of potential risks in radiotherapy-insensitive patients.

However, this study had some limitations. First, the ROI for measuring DECT-derived parameters did not fully reflect the overall characteristics of the lymph nodes. Second, this was a single-center study with no external validation such that DECT parameters may be subjected to contrast agents on a CT scanner, and the impact of differences in scanning and injection protocols and image artifacts in some patients was not validated. Third, we did not explore the correlation between primary tumors, histopathology features, and DECT parameters, which may also contribute to radiotherapy sensitivity prediction.

## Conclusions

In this study, we demonstrated that patients with different nasopharyngeal carcinoma radiotherapy sensitivities have unique DECT imaging parameter characteristics that can be used to predict radiotherapy sensitivity. A visualized nomogram with combined clinical features was constructed, which is a new clinical analysis tool for predicting the radiosensitivity of patients with NPC.

### Electronic supplementary material

Below is the link to the electronic supplementary material.

Supplementary Material 1

Supplementary Material 2

Supplementary Material 5

Supplementary Material 6

Supplementary Material 7

## Data Availability

All data generated or analyzed during this study are included in this article and its supplementary information files.

## Abbreviations

NPC: Nasopharyngeal Carcinoma
DECT: Dual-energy Computed Tomography
CR: Complete Remission
PR: Partial Remission
ROC: Receiver Operating Characteristic
IC: Iodine Concentration
AUC: LAS contraction
AF: Area Under the Curve
OS: Overall Survival
MRI: Magnetic Resonance Imaging
VMIs: Virtual Monoenergetic Images
CCRT: Concurrent Chemoradiotherapy
EBV: EpsteineBarr Virus
BMI: Body Mass Index
GP: Gemcitabine and Platinum
TP: Taxane and Platinum
UNP: Upper Neck Group
MNP: Middle Neck Group
LNP: Lower Neck Group
LD: The Longestdimension of Lymph Nodes
MRI: Magnetic Resonance Imaging
SD: The Shortest Dimension of Lymph Nodes
NIC: Normalized Iodine Concentration
Mix-0.6: Linear Blending Images with a Blending Ratio of 0.6
λHU: Slope of the sPectral Hounsfeld Unit Curve
Z_eff_: Efective Atomic Number
Rho: Electron Cloud Density
AP: Arterial Phase
VP: Venous Phase
*CI*: Confdence Interval
