# Supplementary material for: Quantitative parameter analysis of pretreatment dual-energy computed tomography in nasopharyngeal carcinoma cervical lymph node characteristics and prediction of radiotherapy sensitivity
Source: Radiat Oncol. 2024 Jun 26;19:81. doi: 10.1186/s13014-024-02468-9 (PMC11200824; doi:10.1186/s13014-024-02468-9)
Supplement: Supplementary file 3 — Supplementary Material 5 [file 13014_2024_2468_MOESM5_ESM.doc]

**Author’s name****:**Zhiru Li^1,2,3+^, Chao Li^4+^ , Liyan Li^5^,Dong Yang^2,3^, Shuangyue Wang^2,3^,Junmei Song^2,3^,Muliang Jiang^5*^,Min Kang^2^,^3*^

1 Department of Oncology, Sichuan Provincial People's Hospital·Qionglai Medical Center Hospital, Chengdu, Sichuan, People’s Republic of China

2 Department of Radiation Oncology, The First Affiliated Hospital of Guangxi

Medical University, Nanning, Guangxi, People’s Republic of China

3 Guangxi Tumor Radiation Therapy Clinical Medical Research Center,

Nanning, Guangxi, People’s Republic of China

4 Department of  Obstetrics and Gynecology, Sichuan Provincial People's Hospital·Qionglai Medical Center Hospital, Chengdu, Sichuan, People’s Republic of China

5 Department of Radiology, The First Affiliated Hospital of Guangxi Medical University, Nanning, Guangxi, People’s Republic of China

+These authors contributed equally to this work.

*These authors are co-corresponding authors

**Corresponding Author:**

Kang Min, Department of Radiation Oncology, The First Affiliated Hospital of

Guangxi Medical University, No. 6, Shuangyong Road, Nanning 530021, Guangxi, People’s Republic of China.

**Email:** kangmin@gxmu.edu.cn
