# Supplementary material for: Quantitative parameter analysis of pretreatment dual-energy computed tomography in nasopharyngeal carcinoma cervical lymph node characteristics and prediction of radiotherapy sensitivity
Source: Radiat Oncol. 2024 Jun 26;19:81. doi: 10.1186/s13014-024-02468-9 (PMC11200824; doi:10.1186/s13014-024-02468-9)
Supplement: Supplementary file 4 — Supplementary Material 6 [file 13014_2024_2468_MOESM6_ESM.doc]

**Lymph node characteristics and subgroup analysis of DECT parameters**

| NICAP(N stage) | N2(13.25±4.47) | N3(15.64±5.46) |
| --- | --- | --- |
| N1(18.86±9.51) | 5.603* | 3.217* |
| N2(13.25±4.47) |  | 2.385* |
| λHUAP(N stage) | N2(1.94±0.64) | N3(2.18±0.74) |
| N1(2.61±0.94) | .668* | .428* |
| N2(1.94±0.64) |  | 0.23949 |
| 70keVAP(N stage) | N2(77.97±13.59) | N3(82.39±17.99) |
| N1(88.74±22.53) | 10.769* | 6.349 |
| N2(77.97±13.59) |  | 4.420 |
| ICVP(N stage) | N2(2.31±0.47) | N3(2.31±0.55) |
| N1(2.52±0.52) | 0.211 | .215* |
| N2(2.31±0.47) |  | 0.005 |
| NICAP(Lymph node location) | MNP(13.71±5.29) | LNP(13.36±6.85) |
| UNP(16.42±6.18) | 2.719* | 3.065* |
| MNP(13.71±5.29) |  | 0.34643 |
| λHUAP(Lymph node location) | MNP(1.93±0.77) | LNP(1.88±0.88) |
| UNP(2.29±0.75) | .356* | .414* |
| MNP(1.93±0.77) |  | 0.057 |
| 70keVAP(Lymph node location) | MNP(76.41±19.38) | LNP(72.61±24.71) |
| UNP(84.95±16.76) | 8.538* | 12.341* |
| MNP(76.41±19.38) |  | 3.801 |
| NICVP(Lymph node location) | MNP(39.82±9.07) | LNP(36.95±10.99) |
| UNP(43.62±9.32) | 3.807* | 6.679* |
| MNP(39.82±9.07) |  | 2.871 |
| ICVP(Lymph node location) | MNP(2.20±0.58) | LNP(2.05±0.55) |
| UNP(2.39±0.52) | .195* | .341* |
| MNP(2.20±0.58) |  | 0.145 |
| Mix-0.6VP(分区) | MNP(83.53±17.42) | LNP(78.62±18.34) |
| UNP(90.21±13.61) | 6.680* | 11.594* |
| MNP(83.53±17.42) |  | 4.913 |
| NICAP(LD) | 1-1.5cm(16.67±7.26) | ＜1cm(14.86±5.74) |
| ＞1.5cm(15.27±4.98) | 1.394 | 0.413 |
| 1-1.5cm(16.67±7.26) |  | 4.913 |
| Mix-0.6VP(LD) | 1-1.5cm(88.53±13.97) | ＜1cm(84.76±16.71) |
| ＞1.5cm(90.02±15.09) | 1.494 | 5.260* |
| 1-1.5cm(88.53±13.97) |  | 3.765 |
| 70keVAP(SD) | 1-1.5cm(83.24±19.27) | ＜1cm(78.91±19.42) |
| ＞1.5cm(84.33±16.05) | 1.086 | 5.421 |
| 1-1.5cm(83.24±19.27) |  | 4.334 |
| Mix-0.6VP(SD) | 1-1.5cm(88.35±14.13) | ＜1cm(84.76±16.71) |
| ＞1.5cm(90.45±14.96) | 2.101 | 5.686* |
| 1-1.5cm(88.35±14.13) |  | 3.585 |
