# Supplementary material for: Quantitative parameter analysis of pretreatment dual-energy computed tomography in nasopharyngeal carcinoma cervical lymph node characteristics and prediction of radiotherapy sensitivity
Source: Radiat Oncol. 2024 Jun 26;19:81. doi: 10.1186/s13014-024-02468-9 (PMC11200824; doi:10.1186/s13014-024-02468-9)
Supplement: Supplementary file 5 — Supplementary Material 7 [file 13014_2024_2468_MOESM7_ESM.pdf]

FIRST AFFILIATED HOSPITAL of GUANGXI MEDICAL UNIVERSITY
ETHICAL REVIEW COMMITTEE
Approval Notice

Approval Number: 2023-E329-01

Title: Dual-energy computed tomography quantitative parameter analysis of nasopharyngeal carcinoma cervical lymph node characteristics and prediction of radiotherapy sensitivity: A prospective study

Research Contents: Background and purpose: Treatment efficacy may differ among patients with nasopharyngeal carcinoma (NPC) at a similar tumor–node–metastasis stage. Moreover, end-of-treatment tumor regression is a reliable indicator of treatment sensitivity. This study aimed to investigate whether quantitative dual-energy computer tomography (DECT) parameters can predict the sensitivity of neck-lymph node radiotherapy in patients with NPC.

Materials and methods

Patient population

This study was performed in accordance with the tenets of the Declaration of Helsinki (revised in 2013) and was approved by the Medical Ethics Committee of the First Affiliated Hospital of Guangxi Medical University. All patients signed an informed consent form after receiving a detailed explanation of the research. Between September 2021 and December 2022, 98 patients newly diagnosed with nasopharyngeal carcinoma underwent pretreatment DECT. The study included patients (I) with pathologically confirmed NPC; (II) who had not received radiotherapy or chemotherapy before surgery; and (III) without a history of iodine allergy or hyperthyroidism symptoms. The exclusion criteria were as follows: (1) incomplete clinical data; (2) poor image quality that could not be qualitatively or quantitatively analyzed; and (3) a history of tuberculosis, other head and neck malignancies, or lymphoma.

All patients underwent dual-energy DECT and MRI scans 1–3 days before treatment with radical intensity-modulated radiation therapy and concurrent ± induction chemotherapy. Radiotherapy was administered according to Reports 50 and 62 of the International Commission on Radiation Units and Measurements. Treatment doses included PTVnx and PTVRPN (68–74 GY), PTVND (66–70 GY), PTV1 (60–66 GY), and PTV2 (50–56 GY). Five fractions/week and a total of 30–33 fractions were administered. All chemotherapy regimens were platinum-based (80–100 mg/m2) and administered once every 3 weeks, including radiotherapy alone, concurrent chemoradiotherapy (CCRT), and CCRT after induction chemotherapy. Clinical and pathological data, including age, sex, body mass index, TNM staging, comorbidities, Epstein–Barr virus (EBV) DNA levels, radiation and chemotherapy status, and lymph node characteristics, were collected.

This study aimed to investigate whether quantitative DECT parameters can predict cervical-lymph node radiotherapy sensitivity to NPC, construct a nomogram by combining clinical and pathological factors with quantitative DECT parameters, evaluate the robustness of this new clinical predictive model, and provide new ideas for clinical diagnosis and treatment of NPC.

Applicant: Min Kang

Application Department: Department of Radiation Oncology

Date of Application: July 08, 2023

Date of Approval: July 11, 2023

Conclusion: This paper fully considered and protected the rights and interests of the study objects. It meets the criteria of Ethical Review Committee. The Medical Ethics Committee of First Affiliated Hospital of Guangxi Medical University has approved the protocol.

Signature: ______________________

(Vice) Director of Ethical Review Committee

First Affiliated Hospital of Guangxi Medical University

Date: __July 11, 2023__
